# The Influence of Overweight and Obesity on Treatment Response in Juvenile Idiopathic Arthritis

**DOI:** 10.3389/fphar.2019.00637

**Published:** 2019-06-12

**Authors:** Teresa Giani, Salvatore De Masi, Ilaria Maccora, Francesca Tirelli, Gabriele Simonini, Martina Falconi, Rolando Cimaz

**Affiliations:** ^1^Rheumatology Unit, A. Meyer Children’s Hospital, Florence, Italy; ^2^Department of Medical Biotechnology, University of Siena, Siena, Italy; ^3^Clinical Trial Office, A. Meyer Children’s Hospital, Florence, Italy; ^4^Rheumatology Unit, A. Meyer Children’s Hospital, University of Florence, Florence, Italy; ^5^Technical-Scientific Secretariat of the Paediatric Regional Ethics Committee, Florence, Italy; ^6^Department of Clinical Sciences and Community Health, University of Milan, Milan, Italy

**Keywords:** obesity, overweight, body mass index, juvenile idiopathic arthritis, disease-modifying antirheumatic drugs, biologics

## Abstract

**Background:** There is evidence that obesity could be a risk factor for the severity and response to treatment in adult patients with rheumatoid arthritis (RA) due both to the mechanical effect of overweight and to the potential pro-inflammatory effects of cytokines produced by adipose tissue.

**Objectives:** To evaluate the role of overweight and obesity in a cohort of young patients with juvenile idiopathic arthritis (JIA) in terms of incidence, disease activity, outcome, and response to treatments.

**Methods:** This single-center retrospective cohort study evaluated 110 children affected by JIA under treatment with conventional disease-modifying antirheumatic drugs (DMARDs) and biologic agents. Body mass index (BMI) categories of 5–84th (normal weight), 85–94th (overweight), and ≥95th (obese) percentile were used. Patients with systemic JIA, uveitis, chronic comorbidities, or under other potentially confounding systemic treatments were excluded. Uni- and multivariate analyses were performed.

**Results:** One hundred and ten JIA patients (polyarticular *n* = 50, oligoarticular *n* = 38, psoriatic *n* = 12, enthesitis-related arthritis *n* = 8, undifferentiated *n* = 2) were enrolled in the study, 75% girls and 25% boys. The mean age at treatment onset was 6.09 years. Baseline BMI was ≥5th and ≤84th percentile in 80 patients, 85–94th in 27, and ≥95th in 3.

We did not observe a significant association between BMI and erythrocyte sedimentation rate (ESR), C-reactive protein (CRP), or number of active joints at baseline, while involvement of the joints of lower limbs was significantly greater ( *p* = 0.025) in overweight/obese patients. However, a trend toward lower remission rates and higher number of relapses, both after DMARDs and biologics, in patients with higher BMI was observed.

**Conclusion:** This study focuses on the relationship between overweight/obesity and JIA. A significant correlation between obesity and a greater involvement of the joints of the lower limbs at baseline was demonstrated. Furthermore, our data suggest that obesity could negatively influence the course of the disease as well as treatment response.

## Introduction

Obesity is a medical condition characterized by an excess in body fat accumulation associated with a potentially negative impact on health (Fact Sheet on Obesity and Overweight, 2006). Body mass index (BMI, weight/height^2^, kg/m^2^) is the most widely used method to define obesity and overweight, although it does not take into account body composition and the share of adiposity (Cole et al., 2000). Referring to the US Centers for Disease Control and Prevention (CDC) BMI-for-age growth chart, obesity is defined among subjects aged 2 to 19 years in the presence of a BMI at or above the sex-specific 95th percentile, while overweight is defined as a BMI at or above the 85th percentile and below the 95th (Krebs et al., 2007; Katzmarzyk et al., 2015).

The prevalence of overweight and obesity in European countries accounts for 53.1%, and in the pediatric age, it is about 20%, ranging from 40% in Southern Europe to less than 10% in Northern European countries (Ahrens et al., 2014; Marques et al., 2018).

An increased accumulation of body fat represents a significant risk factor for metabolic complications and a modifiable variable for a number of chronic diseases (Finucane et al., 2011). A wealth of studies both in mice and in humans have shown increased levels of pro-inflammatory cytokines in adipose tissues, including TNF-α, interleukin 6 (IL-6), IL-1β, and several other inflammatory proteins (Hotamisligil et al., 1995; Berg and Scherer, 2005).

Recently, evidence has emerged in favor of the potential negative influence of obesity on rheumatoid arthritis (RA) disease progression, disease severity, and treatment response (Klaasen et al., 2011; Gremese et al., 2013; Heimans et al., 2013; Ellerby et al., 2014; Sandberg et al., 2014; Liu et al., 2017). Liu et al. (2017) in their meta-analysis documented the unfavorable effect of obesity on achieving remission in RA and its impact on disease activity and patient-reported outcomes during therapy. Conversely, only few data have been published about the effect of obesity on juvenile idiopathic arthritis (JIA).

Thus, the goal of our study was to investigate the relationship between BMI and JIA in terms of baseline disease activity and response to classic disease-modifying antirheumatic drugs (DMARDs) and biological agents.

## Materials and Methods

A retrospective study was conducted with an analysis of the medical records of children with JIA classified according to International League of Associations for Rheumatology (ILAR) criteria that were seen in the Pediatric Rheumatology Unit of Anna Meyer Children Hospital between January 2009 and January 2017 (Petty et al., 2004). Only patients who needed systemic drugs were selected. Arthritis related with uveitis and systemic JIA subtypes were considered exclusion criteria.

Demographic data, age at disease onset, anthropometric measurements, disease duration, laboratory data, joint involvement, and medications including DMARDs and biologic drugs were extracted from charts and inserted into a dedicated database. Patients were usually evaluated every 3 months with a thorough physical examination inclusive of assessment of the number and the type of active joints (swelling or both tenderness and limited range of motion) and laboratory investigations.

Height was measured with a wall-mounted stadiometer to the nearest 0.1 cm, while for weight, a calibrated beam balance platform scale to the nearest 0.1 kg was used. BMI was calculated as weight in kilograms divided by height in square meters. BMI-for-age percentiles were computed using the CDC reference values. Normal BMI was defined as the 5th percentile to less than the 85th percentile, overweight as the 85th to less than the 95th percentile, and obese as equal to or greater than the 95th percentile (Kuczmarski et al., 2002).

Relevant laboratory variables included erythrocyte sedimentation rate (ESR), and C-reactive protein (CRP). Clinically inactive disease status was defined as no joints with active disease, an ESR of ≤20 mm/h, and CRP ≤0.5 mg/dl.

The data were analyzed using the person time method. Patients on treatment with first-line drugs (DMARDs) were followed from the start of treatment up to the date of remission or last follow-up. The remission rates for person-months were therefore calculated. The rates of relapse were calculated as the number of relapse (first occurrence) after remission on person-months at risk. The amount of person-months was the sum of months at risk from the date of remission to the date of relapse or of the last follow-up. For subjects eligible for treatment with biological drugs, remission and relapse post-remission rates were calculated using the same method. We performed univariate and multivariate analysis and included in the models clinically and statistically relevant variables as covariate. All analyses were performed with the STATA 11 software.

Since we used deidentified data, according to national regulation, no ethics committee approval or informed consent was needed.

## Results

Demographic and clinical data are reported in **Table 1**. One hundred and ten JIA patients (polyarticular *n* = 50, oligoarticular *n* = 38, psoriatic *n* = 12, enthesitis-related arthritis *n* = 8, undifferentiated *n* = 2) were enrolled in the study, 75% girls and 25% boys. The mean age at conventional DMARDs treatment onset was 6.09 years. Baseline BMI was included between the 5th and 84th percentile in 80 patients, 85–94th in 27, and ≥95th in 3.

**Table 1:** Baseline characteristics of the population.

Variables		BMI 5–84th percentile	BMI ≥85th percentile	Total	*p*
**Age (years) at enrollment**	Mean (SD)	6.0 (4.04)	6.2 (3.4)	6.0 (3.8)	0.7782
**Age at puberty (years)**	Mean (SD)	11.4 (1.7)	10.5 (1.4)	11.1 (1.7)	0.1197
**Gender**	Females	57 (71.2%)	26 (86.6%)	83 (75.4%)	0.094
	Males	23 (28.7%)	4 (13.3%)	27 (24.5%)	
**ESR (mm/h)**	0–19	23 (30.2%)	6 (21.4%)	29 (27.8%)	0.373
	≥ 20	53 (69.7%)	22 (78.5%)	75 (72.1%)	
**CRP (mg/dl)**	0.0–0.49	32 (43.2%)	8 (28.5%)	40 (39.2%)	0.176
	≥ 0.5	42 (56.7%)	20 (71.4%)	62 (60.7%)	
**Number of active joints**	0–4	49 (61.2%)	13 (43.3%)	62 (56.3%)	0.092
	≥ 5	31 (38.7%)	17 (56.6%)	48 (43.6%)	
**Number of active joints in lower limbs**	0–4	66 (88%)	18 (69.2%)	84 (83.1%)	0.028*
	≥ 5	9 (12%)	8 (30.7%)	17 (16.8%)	
**JIA category**	ERA	6 (7.5)	2 (6.6)	8 (7.2)	0.645
	Undifferentiated	1 (1.2)	1 (3.3)	2 (1.8)	
	Oligoarticular	31 (38.7)	7 (23.3)	38 (34.5)	
	Polyarticular RF neg	33 (41.2)	16 (53.3)	49 (44.5)	
	Polyarticular RF pos	1 (1.2)	0 (0.0)	1 (0.9)	
	Psoriatic	8 (10.0)	4 (13.3)	12 (10.9)	

*Statistical significance.

At baseline, ESR and CRP values were increased, although not significantly, in overweight/obese compared with healthy weighted individuals (*p* = 0.373 and 0.176 for ESR and CRP, respectively). The number of active lower limb joints was significantly lower (*p* = 0.028) in healthy weighted than in overweight/obese individuals (**Table 1**).

Sixty-nine patients out of 110 achieved remission on conventional DMARDs. The remission rate was 93.88/1000 person-months, with higher remission rates in healthy weighted children (98.87) than in obese children (83.58). At univariate analysis, however, the difference did not reach statistical significance. The hazard ratio (HR) in obese children compared with healthy weighted children was 0.8, CI 95% 0.46–1.40. An abnormal CRP value was the only covariate influencing the remission rate, close to statistical significance (HR 1.79, CI 95% 0.98–3.26) (**Table 2**).

**Table 2:** Remission with classical DMARDs.

Variables	*n* events	Person-months	Incidence rate × 1000 person-months	Hazard ratio (CI 95%)
**All patients**	69	734.9	93.8	
**BMI**				
5–84th percentile	48	485.4	98.8	1
≥85th percentile	17	203.4	83.5	0.8 (0.46–1.40)
**Age at puberty (years)**				
0–10	9	103.2	87.2	1
≥11	13	171.7	75.7	0.74 (0.3–1.84)
**ESR (mm/h)**				
0–19	15	154.2	97.2	1
≥20	50	498.8	100.2	1.23 (0.67–2.27)
**CRP (mg/dL)**				
0.0–0.49	20	246.98	80.98	1
≥0.50	43	391.64	109.80	1.79 (0.98–3.26)
**Number of active joints**				
0–4	39	371.2	105.0	1
≥5	30	363.6	82.4	0.80 (0.48–1.31)
**Number of active joints in lower limbs**				
0–4	52	576.3	90.2	1
≥5	11	109.5	100.4	1.28 (0.65–2.5)

Incidence rate × 1,000 person-months and hazard ratio. Univariate analysis.

The multivariate analysis showed a significant effect of elevated CRP value on the remission rate (HR 2.21, CI 95% 1.12–4.35). Lower remission rates were observed in children with an overweight/obese BMI and a higher number of active joints (more than five) at baseline as well as in patients with increased values of ESR and CRP compared to normal values (HR 1.77, CI 95% 0.62–5.06 and HR 1.72, CI 95% 0.75–3.98, for ESR and CRP, respectively), although without a statistical significance (**Tables 3** and **4**). Thirty-five patients out of 69 relapsed after remission, with a relapse rate of 13.40/1000 person-months. The relapse rate was slightly higher in obese children (15.79) than in healthy weighted children (13.37). The HR was 1.25, CI 95% 0.6–2.6.

**Table 3:** Remission with classic (DMARDs).

Variables		Hazard ratio	(CI 95%)
**BMI (percentile)**	0–84th	1	
	≥85th	0.77	0.42–1.43
			
**CRP (mg/dL)**	0.0–0.49	1	
	≥0.50	2.21	1.12–4.35
			
**Number of active joints**	0–4	1	
	≥5	0.67	0.37–1.20

Multivariate analysis. Cox regression.

**Table 4:** Relapse post-remission with classic DMARDs.

Variables	*n* events	Person-months	Incidence rate × 1000 person-months	Hazard ratio (CI 95%)
**All patients**	35	2611.0	13.4	
**BMI**				
0–84th percentile	22	1645.0	13.3	1
≥85th percentile	11	696.8	15.7	1.25 (0.6–2.6)
**Age at puberty (years)**				
0–10	8	268.7	29.7	1
≥11	9	538.8	16.7	0.58 (0.22–1.50)
**ESR (mm/h)**				
0–19	4	515.7	7.7	1
≥20	29	1925.2	15.0	1.77 (0.62–5.06)
**CRP (mg/dL)**				
0.0–0.49	7	799.01	8.76	1
≥0.50	26	1607.34	16.18	1.72 (0.75–3.98)
**Number of active joints**				
0–4	19	1407.9	13.5	1
≥5	16	1203.1	13.3	0.92 (0.47–1.82)
**Number of active joints in lower limbs**				
0–4	27	1910.8	14.1	1
≥5	5	500.3	9.9	0.68 (0.26–1.78)

Incidence rate × 1000 person-months and hazard ratio. Univariate analysis.

The Cox regression showed a higher HR of relapse rate in overweight/obese children compared to healthy weighted children, and in children with abnormal values of CRP compared to those with normal values. However, all HR estimates did not reach statistical significance (**Table 5**).

**Table 5:** Relapse post-remission with classic DMARDS.

Variables		Hazard ratio	(CI 95%)
**BMI (percentile)**	0–84th	1	
	≥85th	1.35	0.61–2.98
			
**CRP (mg/dL)**	0.0–0.49	1	
	≥0.50	1.90	0.80–4.48
			
**Number of joints involved**	0–4	1	
	≥5	0.73	0.34–1.57

Multivariate analysis. Cox regression.

Forty-eight patients required a second-line treatment: 27 etanercept (13 in overweight/obese), 19 adalimumab (5 in overweight/obese), and 2 abatacept (all in healthy weighted children).

Remission rate with second-line therapy was 122.12/1,000 person-months, higher in healthy weighted children (146.21) than in obese children (87.45). The HR was 0.61, CI 95% 0.31–1.20.

Remission rates were lower in individuals with several active joints when compared with those with few active joints (>4 vs. 0–4). The corresponding HR were 0.59, CI 95% 0.32–1.08 for more than 4 active joints vs. 0–4, and 0.46, CI 95% 0.19–1.13 (>4 vs. 0–4 joints) considering only lower limbs (**Table 6**). The multivariate analysis confirmed the results of the univariate calculation (**Table 7**).

**Table 6:** Remission with second-line therapy (biologics).

Variables	*n* events	Person-months	Incidence rate × 1000 person-months	Hazard ratio (CI 95%)
**All patients**	43	352.1	122.1	
**BMI**				
0–84th percentile	27	184.6	146.2	1
≥85th percentile	14	160.1	87.4	0.61 (0.31–1.20)
**Age at puberty (years)**				
0–10	10	114.1	87.5	1
≥11	13	91.3	142.2	1.56 (0.66–3.68)
**ESR (mm/h)**				
0–19	13	96.3	134.9	1
≥20	22	216.5	101.6	0.81 (0.40–1.63)
**CRP (mg/dl)**				
0.0–0.49	20	155.93	128.26	1
≥0.50	15	155.04	96.75	0.76 (0.38–1.51)
**Number of active joints**				
0–4	20	119.3	167.5	1
≥5	23	232.7	98.8	0.59 (0.32–1.08)
**Number of active joints in lower limbs**				
0–4	31	200.0	154.9	1
≥5	6	67.9	88.3	0.46 (0.19–1.13)

Incidence rate × 1000 person-months and hazard ratio. Univariate analysis.

**Table 7:** Remission with second-line therapy (biologics).

Variables		Hazard ratio	(CI 95%)
**BMI (percentile)**	0–84th	1.0	
	≥85th	0.49	(0.19–1.24)
**Age at puberty (years)**	0-10	1.0	
	≥11	1.93	(0.77–4.80)
**Number of active joints**	0–4	1.0	
	≥5	0.49	(0.20–1.20)

Multivariate analysis. Cox regression.

## Discussion

We investigated the negative effect exerted by obesity on JIA as suggested by data on adults affected by RA. Although JIA shares some features with RA, they should be considered distinct diseases. JIA represents the most common rheumatic disease of childhood with a prevalence of 16–150 cases per 100,000 children (Prakken et al., 2011). Regardless of the differences in the underlying pathogenesis of the various types of JIA, the immunopathology involves a predominant abnormality of the adaptive immune system with a consistent overproduction of pro-inflammatory cytokines, many of which, such as TNF-α, IL-1, and IL-6, have been shown to be increased also in the presence of adipose expansion.

We observed a very small number of obese subjects in our cohort of patients; therefore, we divided it into two groups, one comprising children with a “healthy weight” (BMI < 85th percentile) and the other comprising children with an “increased weight” (BMI ≥ 85th percentile). Overweight/obesity rate in our study population is 27%, reflecting the pediatric prevalence of this condition in our country. We also excluded patients affected by systemic subtype, for its clinical heterogeneity compared to the other JIA subtypes, and patients with uveitis, which may require further treatments despite a good arthritis control, to avoid confounding factors.

At baseline, we observed a negative influence of obesity/overweight on disease activity. Our JIA overweight/obese children showed a tendency to have a greater number of active joints, in particular those of lower limbs, and higher values of ESR and CRP compared to healthy weight subjects.

Few data are available about the impact of obesity on JIA. A first study by Pelajo et al. analyzed 154 JIA subjects, 18% of whom were obese and 12% were overweight; no association was found between obesity and disease activity in terms of Juvenile Arthritis Disease Activity Score 27, physician’s assessment of disease activity, parent’s assessment of child’s well-being, CRP, ESR, and number of active joints (Pelajo et al., 2012).

On the other hand, Amine et al. (2011) in their series of 58 Moroccan JIA patients, characterized by high BMI prevalence (41.4% overweight and 22.4% obese), observed a correlation between overweight and functional limitation and severe joint pain.

In 2016, Makay et al. studied 72 patients with enthesitis-related arthritis, 27% of whom have a BMI ≥ 85th percentile. In subjects with increased BMI, they observed a higher frequency of tarsitis and ankle arthritis at baseline, while sacroiliac, hip, and entheses involvement as well as ESR values were similar to those with a BMI < 85th percentile (Makay et al., 2016).

Compared to adult RA, the correlation between disease activity and obesity seems to be less evident in JIA. Furthermore, data regarding the influence of BMI in treatment responses and outcome are still very poor in the pediatric age. In RA, obesity appears to be a strong predictor of worse treatment responses, decreasing the chances of achieving good disease control during the early phase, in which most of the patients receive DMARDs (Sandberg et al., 2014). Some recent studies have shown that the effect of TNF blockage also appears to be impaired in obese RA patients, while no significant difference between obese and non-obese were seen in patients treated with abatacept and tocilizumab (Klaasen et al., 2011; Gremese et al., 2013; Iannone et al., 2015; Kim et al., 2016; Shan and Zhang, 2018). The modification in the pharmacokinetics and pharmacodynamics of drugs related to the excess of fat has been hypothesized to influence the response to therapy. On the other hand, obesity may mask early identification of active joints, delaying the beginning of the correct treatment (Barnabe et al., 2014).

In pediatrics, only the study by Makay et al. (2016) evaluated the response to therapy in patients with enthesitis-related arthritis with different BMI, reporting a failure in achieving a clinically inactive disease in children with a higher BMI treated with DMARDs or anti-TNF drugs, suggesting a negative effect of obesity on disease outcome.

In our JIA population, obesity/overweight corresponds to a worse trend in the therapeutic response both to first-line treatments and to anti-TNF drugs, although without reaching statistical significance.

Despite the fact that our data do not identify obesity as an independent risk factor for JIA, they suggest a negative influence on disease activity at baseline and an unfavorable impact on the therapeutic response to conventional DMADs and anti-TNF drugs. These observations may take into consideration weight management with dietary and physical activity interventions as part of the treatment strategy for obese/overweight patients.

Limitations of our study include the fact that few of our patients were obese and that the sample size of our population was relatively small; this may have hampered the achievement of statistical significance of several comparisons. Nonetheless, our study is one of the few that have investigated such factors and deserves further investigations.

## Author Contributions

TG was in charge of project planning and paper writing. FT and IM were in charge of data collection. SM and MF performed the statistical analysis. GS and RC were the coordinators.

## Conflict of Interest Statement

The authors declare that the research was conducted in the absence of any commercial or financial relationships that could be construed as a potential conflict of interest.
